# High expression of p62/SQSTM1 predicts shorter survival for patients with pancreatic cancer

**DOI:** 10.1186/s12885-022-09468-6

**Published:** 2022-03-30

**Authors:** Eva Philipson, Cecilia Engström, Peter Naredi, Johan Bourghardt Fagman

**Affiliations:** 1 Department of Surgery, Institute of Clinical Sciences, Sahlgrenska Academy, University of Gothenburg, Gothenburg, Sweden; 2 Department of Surgery, Sahlgrenska University Hospital, Sahlgrenska, Vita Stråket 12, paviljong plan 2, SE-413 45 Gothenburg, Sweden

**Keywords:** p62/SQSTM1, Survival, Pancreatic cancer

## Abstract

**Background:**

Accumulation of the signal adaptor protein p62 has been demonstrated in many forms of cancer, including pancreatic ductal adenocarcinoma (PDAC). Although data from experimental studies suggest that p62 accumulation accelerates the development of PDAC, the association between p62 protein expression and survival in PDAC patients is unclear.

**Methods:**

Thirty-three tumor specimens from PDAC patients treated by primary surgery were obtained. Immunohistochemical expression of p62, microtubule-associated protein 1A/1B-light chain 3 (LC3), and nuclear factor-erythroid factor 2-related factor 2 (NRF2) in tumor tissue was examined for associations with clinicopathological characteristics and disease-specific survival (DSS).

**Results:**

There was no association between p62 expression and any of the clinicopathological variables. However, high p62 protein expression in tumor cells was significantly associated with shorter DSS (7 months vs. 29 months, *p* = 0.017). The hazard ratio for death in patients with high p62 protein expression in tumor cells was 2.88 (95% confidence interval: 1.17–7.11, *p* = 0.022). In multivariable analysis, high p62 expression was an independent prognostic factor for shorter DSS (*p* = 0.020) when follow up time was more than 5 years. LC3 and NRF2 staining was not associated with survival or other clinicopathological parameters.

**Conclusion:**

Our results show that high p62 protein expression in tumor cells is associated with shorter survival following pancreatic tumor resection. This association supports a role for p62 as a prognostic marker in patients with PDAC treated by primary surgery.

**Supplementary Information:**

The online version contains supplementary material available at 10.1186/s12885-022-09468-6.

## Introduction

The stress-inducible adaptor protein p62/SQSTM1 (hereafter p62) plays important roles in the development of pancreatic cancer [1, 2]. P62 is a multi-domain protein that was originally defined as an autophagy receptor. In autophagy, p62 targets proteins and organelles for lysosomal degradation by linking cargo to microtubule-associated protein 1A/1B-light chain 3 (LC3) in the membrane of autophagosomes. However, p62 also can induce downstream signaling pathways, including NF-κB, mTORC1, and nuclear factor-erythroid factor 2-related factor 2 (NRF2), to influence inflammation, nutrient sensing, and the oxidative stress defense, which all may affect tumorigenesis [3, 4]. Experimental studies using mouse models have shown that accumulation of p62 activates NF-κB and NRF2 signaling and accelerates the development of pancreatic cancer [1, 2, 5]. Although both NRF2 and NF-κB are elevated in human pancreatic cancer [1, 6–8], little is known about the role of p62 accumulation and associations with its downstream pathways in the development of human pancreatic cancer.

Immunohistochemical staining for p62 has been detected in many human cancers including esophageal, gastric, and large intestinal cancers, hepatocellular carcinoma, and pancreatic ductal adenocarcinoma (PDAC) [9, 10], suggesting an association of cancer with p62 accumulation. Expression of p62 also has been linked to tumor grade, distant metastasis [11], and higher risk of metastasis and poor prognosis, particularly in breast cancer [12]. Few studies have examined the expression of p62 in PDAC tissue from patients. Although the available data indicate increased expression of p62 [2, 10], there is little information about the role of p62 accumulation in PDAC prognosis.

To elucidate the relevance of p62 expression in PDAC tissue and its prognostic value, we examined expression of p62 protein in tumor tissue sections from patients with PDAC and analyzed the association between the immunoreactivities and clinicopathological parameters. We also investigated whether an association between p62 protein expression and patient outcomes was independent of LC3 (autophagy) and NRF2 (antioxidant pathway activation).

## Material and methods

All methods were carried out in accordance with SAMPL Guidelines.

### Patients and tumor tissue samples

This study was conducted in accordance with the principles of the Declaration of Helsinki and approved by the Regional Ethical Review Board at the University of Gothenburg, Gothenburg, Sweden (reference number 002–06), and all participants gave written informed consent. Data were analyzed anonymously. Whole pancreatic tumor tissue sections were obtained from 33 patients (15 female/18 male) diagnosed with PDAC who underwent surgical tumor resection in 1998 to 2005 at Sahlgrenska University Hospital, Gothenburg, Sweden. Patients were included in the study based on the availability of enough archived tumor material to prepare high-quality whole tumor tissue sections for IHC analysis at the hospital pathology department. All patients underwent surgery as the primary treatment, and none had received irradiation or chemotherapy before surgery. Clinical information and follow-up data were obtained from medical records. Resection margin status was R0 for all patients, and all tumors were histologically diagnosed as ductal adenocarcinoma and classified according to the pTNM staging system (sixth edition of the AJCC) by the pathology department.

Disease-specific survival (DSS) was defined as the time between surgery and death attributable to PDAC. Three patients were still alive at the time of analysis. Of the 30 patients who died, 22 died because of PDAC. The remaining eight deaths were attributable to cardiovascular causes (*n* = 3), lung cancer (*n* = 1), neuroendocrine cancer (*n* = 1), postoperative complications (*n* = 1), chronic sub-ileus (*n* = 1), and an unknown cause (*n* = 1). The cause of death for each patient was confirmed by data obtained from the National Board of Health and Welfare in Sweden (reference number 35835/2020).

### Immunohistochemistry

Immunohistochemistry was performed on 4-μm sections of formalin-fixed paraffin-embedded pancreatic tumor tissue. After xylene deparaffinization, ethanol dehydration, and antigen retrieval (microwave oven at 500 W for 2 × 5 min in citrate buffer pH 6.0), endogenous peroxidase activity was quenched by incubation in hydrogen peroxide solution (Peroxidazed 1, PX 968, Biocare Medical, Pacheco, CA, USA) for 5 min. To reduce non-specific background, sections were incubated in casein solution (Background Sniper, BS966, Biocare Medical) for 15 min. Then sections were incubated with primary antibodies in dilution buffer (Da Vinci Green Diluent, PD900, Biocare Medical) overnight at 4 °C, followed by incubation with probe (MACH 1 Mouse probe, UP537, Biocare Medical) for 15 min at room temperature (p62 only) and HRP-polymer (MACH 1 Universal HRP-Polymer, MRH538, Biocare Medical) for 30 min at room temperature. Bound peroxidase was visualized by incubation for 1–10 min in a 3,3′-diaminobenzidine (DAB) solution (Betazoid DAB Chromogen, BDB900 diluted in Betazoid DAB Substrate Buffer, DS900, Biocare Medical). Sections were washed in Tris-buffered saline, counterstained with hematoxylin, dehydrated, and mounted. Slides were photographed on an upright Nikon Eclipse E400 light microscope using a DXM1200 camera with ACT-1 version 2.0 software (Nikon, Japan). The following primary antibodies were used: monoclonal mouse anti-human p62/SQSTM1, raised against amino acids 151–440 of p62/SQSTM1 of human origin, clone D-3, (sc-28,359, Santa Cruz Biotechnology, Dallas, TX, USA), dilution 1:100; polyclonal rabbit anti-LC3B, raised against a synthetic peptide corresponding to the amino terminus of LC3B, (#2775, Cell Signaling Technology, Danvers, MA, USA), dilution 1:100; and polyclonal rabbit anti-NRF2, raised against a synthetic peptide corresponding to amino acids 569–588 of human NRF2 (ab31163, Abcam, Cambridge, UK), dilution 1:200. Normal pancreas was used as positive control for p62 and NRF2, and pancreatic tumor tissue containing β islets with endocrine cells was used as positive control for LC3. Negative controls were performed by replacing primary antibodies with matching isotype control antibodies diluted to the same protein concentration as the primary antibody. The following isotype control antibodies were used: for p62, normal mouse IgG1 (sc-3877, Santa Cruz Biotechnology, Dallas, TX, USA) and for LC3 and NRF2, polyclonal rabbit IgG (ab171870, Abcam, Cambridge, UK).

### Scoring of immunohistochemical staining

Tumor tissue samples were scored semi-quantitatively under light microscopy for cytoplasmic and nuclear staining (p62 and NRF2) or cytoplasmic staining (LC3) in a blinded manner without knowledge of pathological and clinical data. A modified labeling score (H score) was calculated as previously described [13]. Separate scoring of the dominant staining intensity and the percentage of positive tumor cells (glandular or abnormal shape, high nuclear-to-cytoplasmic ratio, abnormal nuclei, i.e., pleomorphic, larger, and darker than in normal cells [14]) was performed using 10 high-magnification (× 200) fields per patient, and scores for each field were averaged. The final staining scores were determined by multiplying the percentage of positive tumor cells (0 to 100%) by the dominant staining intensity (0 = no staining, similar to negative control; 1 = weak staining, weaker than the positive control; 2 = intermediate staining, similar to the positive control; and 3 = strong staining, stronger than the positive control). Resulting scores ranged from 0 to 270 [15]. For statistical analysis, p62, LC3, and NRF2 staining scores were classified into two grades with the mean staining score as the cutoff point (high grade ≥ mean; low grade < mean) and for detailed analysis p62 staining was divided into three grades using staining scores 40 and 140 as cutoff points (p62 low ≤40; p62 intermediate > 40 and ≤ 140; p62 high > 140).

### Statistical analyses

Data are presented as medians and ranges (continuous data) or as numbers and percentages (categorical data). Mann–Whitney *U* and Pearson’s chi-square tests were used to determine the association between p62 protein expression in tumor cells and clinicopathological and molecular parameters. The correlations of p62, LC3, and NRF2 expression were evaluated using Spearman’s rank correlation coefficient. DSS was evaluated using Kaplan–Meier survival plots, and differences in survival were tested using log-rank (Mantel–Cox) tests. Univariable Cox proportional hazards regression analysis was used to estimate hazard ratios (HRs) and 95% confidence intervals (95% CIs). Multivariable Cox proportional hazards regression analysis was performed to assess independent prognostic factors for survival using the following covariables: p62, age, sex, tumor stage, differentiation, lymph node metastasis, and adjuvant therapy. All *p* values corresponded to two-sided tests, and *p* values less than 0.05 were considered statistically significant. All statistical analyses were made using either SPSS version 25 (IBM Corp, Armonk, NY, USA) or GraphPad Prism 9 (GraphPad Software, Inc., La Jolla, CA, USA).

## Results

### Patient characteristics

A total of 33 patients with PDAC were included in this study. The clinical information for all cases is summarized in Table 1. The median patient age was 62 years (range, 50–80 years), and the median survival was 22.6 months (range, 1.1–212.2 months) after surgery. Among the 33 patients, 7 had received postoperative adjuvant treatment with gemcitabine.

**Table 1:** Clinicopathological characteristics of the patients in the study cohort

Variable	All cases
Patients, n	33
Median age, years (range)	62 (50–80)
Age, n (%)	
<65 years	20 (60.6)
≥65 years	13 (39.4)
Sex, n (%)	
Female	15 (45.5)
Male	18 (54.5)
Tumor location, n (%)	
Head	30 (90.9)
Others	3 (9.1)
Tumor stage, n (%)	
pT1	3 (9.1)
pT2	13 (39.4)
pT3	14 (42.4)
pT4	3 (9.1)
Differentiation, n (%)	
Low	10 (30.3)
Medium	14 (42.4)
High	5 (15.2)
Data missing	4 (12.1)
Lymph node metastasis, n (%)	
N0	15 (45.5)
N1	18 (54.5)
Adjuvant chemotherapy, n (%)	
No	26 (78.8)
Yes	7 (21.2)

*pT* Pathologic evaluation of tumor specimen; *N0/N1* No presence/presence of regional lymph node metastasis. Adjuvant chemotherapy = postoperative gemcitabine treatment.

### Correlation between p62 protein expression in tumor cells and clinicopathological features

**Fig. 1:**
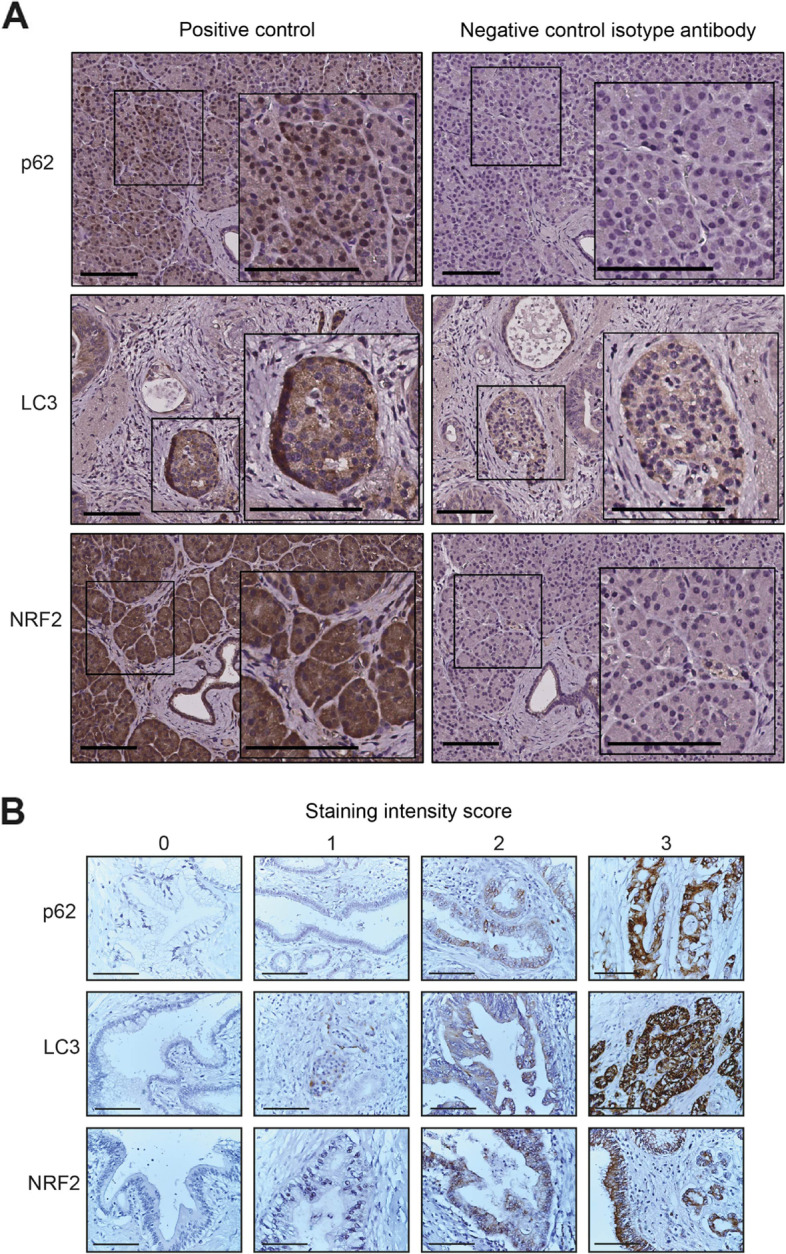
Immunohistochemical staining for p62, LC3, and NRF2. **A** Representative photomicrographs of positive control staining of human normal pancreas (p62 and NRF2) and human pancreatic tumor tissue containing β islets with endocrine cells (LC3). Negative controls were stained with matching isotype control antibodies. **B** Representative cases illustrating the scores based on immunostaining intensity. Scale bars, 100 μm (brown: positive antibody staining, blue: hematoxylin for nuclei staining)

To assess the protein expression of p62, LC3, and NRF2 in pancreatic tumor cells, we performed immunohistochemistry on sections of whole pancreatic tumor tissue from the 33 patients. p62 and NRF2 were expressed in the cytoplasm and in the nucleus. LC3 was mainly expressed in the cytoplasm (Fig. 1A). Positive staining was scored according to the dominant intensity (Fig. 1B) and the percentage of positive tumor cells. The two scores were combined into a staining score.

**Fig. 2:**
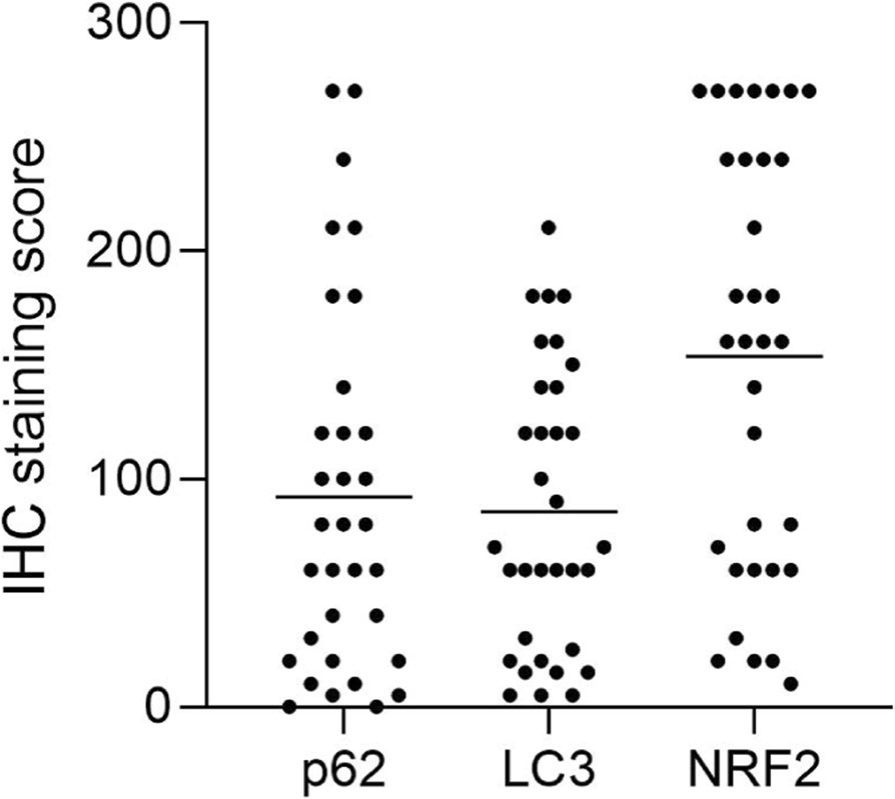
Quantification of immunostaining for p62, LC3, and NRF2 in pancreatic tumors. Scatter plots showing the immunohistochemical staining scores (staining intensity × percentage of positive tumor cells). Lines indicate means, and circles represent individual patients

The mean staining scores of p62, LC3, and NRF2 expression were 92, 86, and 154, respectively (Fig. 2). There was no significant correlation between p62 and LC3 (r_s_ = 0.325, *p* = 0.065), between p62 and NRF2 (r_s_ = 0.117, *p* = 0.518), or between LC3 and NRF2 (r_s_ = 0.045, *p* = 0.805).

**Fig. 3:**
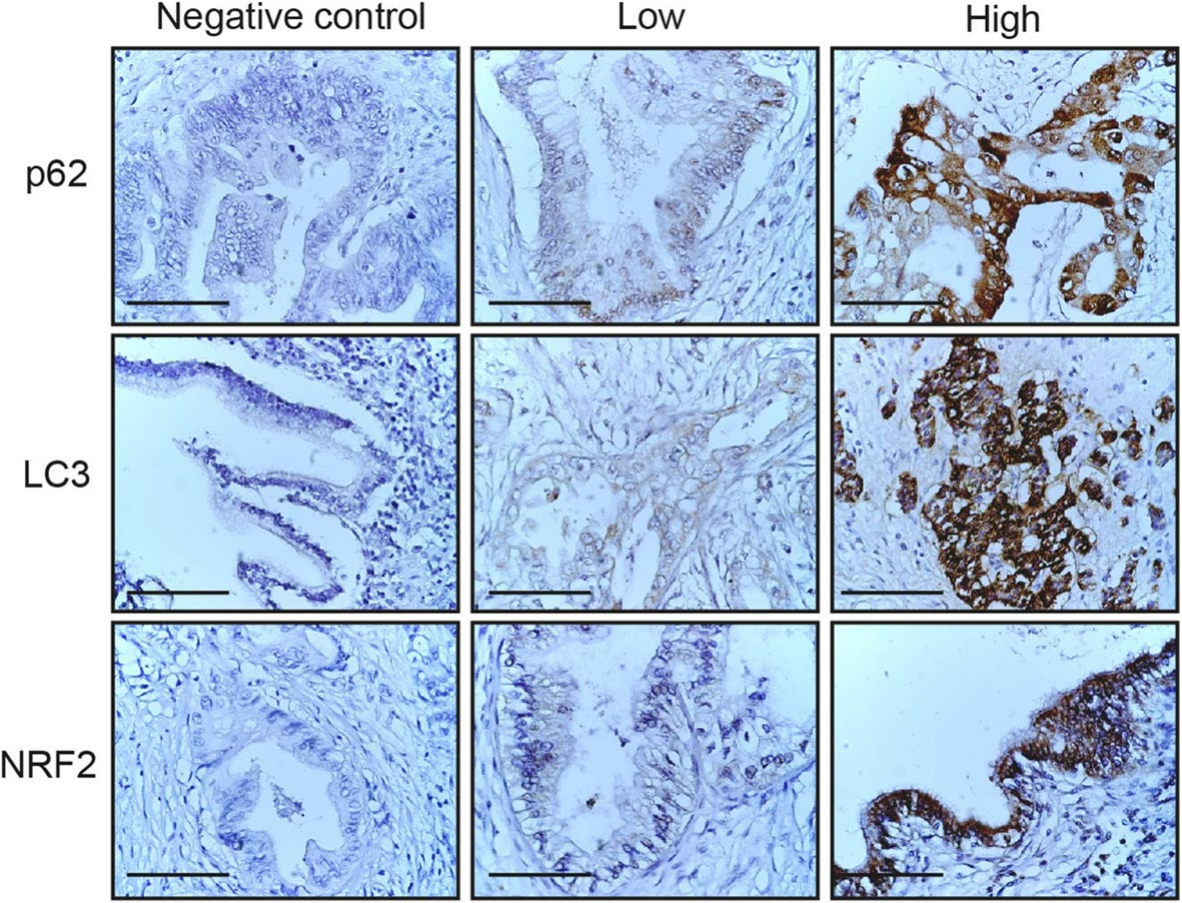
Immunohistochemical staining for p62, LC3, and NRF2 in whole pancreatic tumor tissue sections. Representative areas of pancreatic cancers stained for p62, LC3, and NRF2 and degree of staining scored as low or high. Negative controls were stained with matching isotype control antibodies. Scale bars, 100 μm (brown: positive antibody staining; blue: hematoxylin for nuclei staining)

Using the mean staining score as a cutoff point, we classified the PDAC samples into two grades: low grade and high grade (Fig. 3).

We found no significant correlations between p62 protein expression in tumor cells and clinicopathological characteristics (Table 2).

**Table 2:** Relationship between p62 expression and clinicopathological variables

Variable	p62 low	p62 high	*p*^a^
Patients, n (%)	19 (57.6)	14 (42.4)	0.255^b^
Median age, years (range)	62 (50–76)	62.5 (52–80)	0.733^b^
Age, n (%)			
<65 years	12 (63.2)	8 (57.1)	
≥65 years	7 (36.8)	6 (42.9)	0.727
Sex, n (%)			
Female	7 (36.8)	8 (57.1)	
Male	12 (63.2)	6 (42.9)	0.247
Tumor location, n (%)			
Head	17 (89.5)	13 (92.9)	
Others	2 (10.5)	1 (7.1)	0.738
Tumor stage, n (%)			
pT1	2 (10.5)	1 (7.1)	
pT2	8 (42.1)	5 (35.7)	
pT3	9 (47.4)	5 (35.7)	
pT4	0 (0)	3 (21.4)	0.211
Differentiation, n (%)			
Low	7 (36.8)	3 (21.4)	
Medium	7 (36.8)	7 (50.0)	
High	3 (15.8)	2 (14.3)	0.617
Data missing	2 (10.5)	2 (14.3)	
Lymph node metastasis, n (%)			
N0	10 (52.6)	5 (35.7)	
N1	9 (47.4)	9 (64.3)	0.335
Adjuvant chemotherapy, n (%)			
No	14 (73.7)	12 (85.7)	
Yes	5 (26.3)	2 (14.3)	0.403
LC3, n (%)			
Low	13 (68.4)	5 (35.7)	
High	6 (31.6)	9 (64.3)	0.062
NRF2, n (%)			
Low	9 (47.4)	5 (35.7)	
High	10 (52.6)	9 (64.3)	0.503

^a^Chi-square test, except ^b^Mann–Whitney U test. Low, staining grade low; high, staining grade high; *pT* Pathologic evaluation of tumor specimen, *N0/N1* No presence/presence of regional lymph node metastasis. Adjuvant chemotherapy = postoperative gemcitabine treatment

### High expression of p62 in tumor cells is a prognostic factor for survival in patients with resected PDAC

**Fig. 4:**
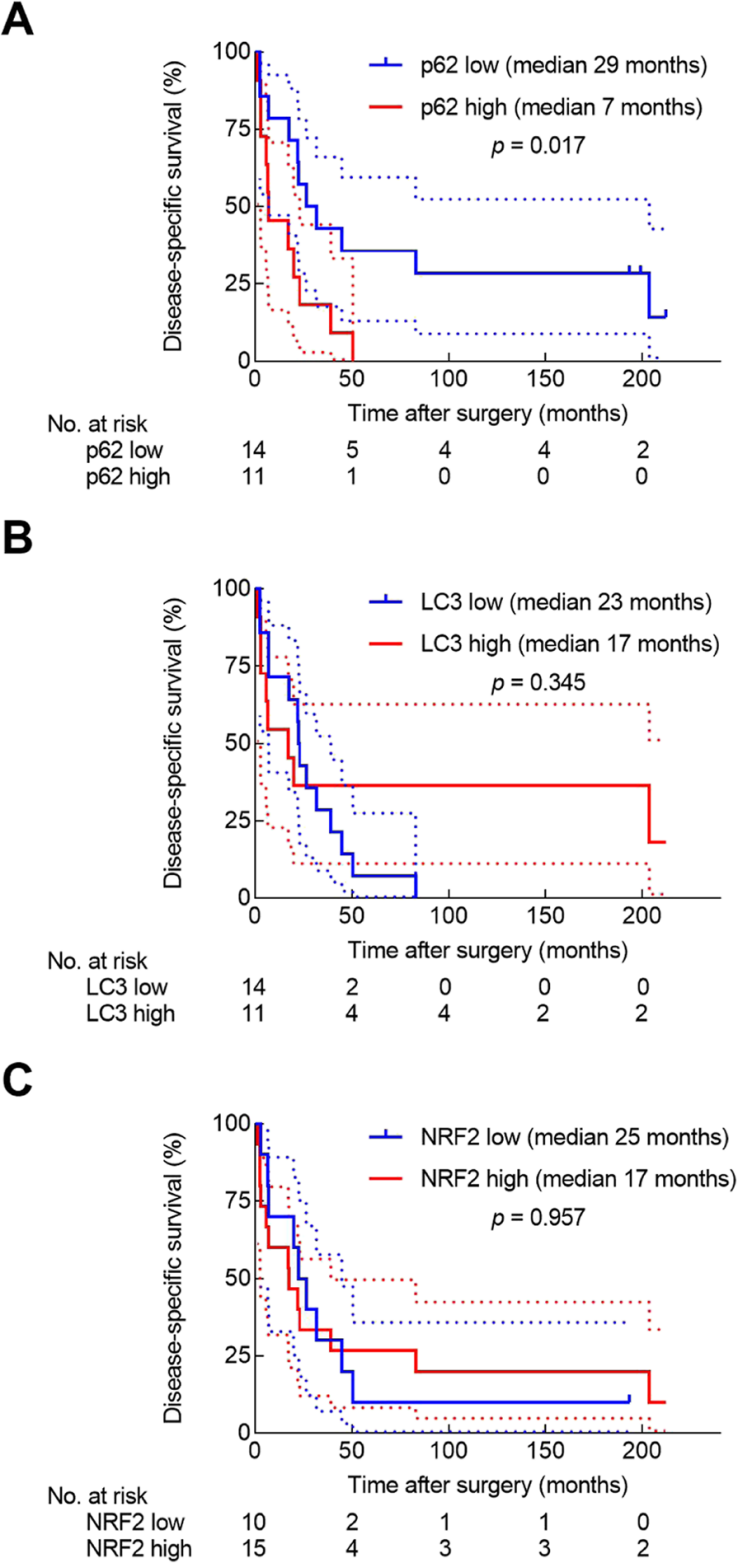
p62 expression correlates with shorter survival in patients with pancreatic cancer. Kaplan–Meier curves of disease-specific survival (*n* = 25) according to p62 (**A**), LC3 (**B**), and NRF2 (**C**). Solid lines represent the survival curves, and the dotted lines indicate the 95% confidence intervals for the survival curves

To examine the prognostic impact of p62, LC3, and NRF2 on survival outcome, we analyzed DSS according to p62, LC3, and NRF2 protein expression in tumor cells. Kaplan–Meier survival analysis using the log-rank test showed that high p62 expression in tumor cells was significantly associated with shorter DSS (Fig. 4A), whereas high LC3 or NRF2 was not (Fig. 4B, C).

The remaining clinicopathologic factors did not correlate with survival (Table 3).

**Table 3:** Disease-specific survival according to clinicopathological factors

Variable	Cases	Deaths	Median survival (months)	95% CI	Log-rank test (*p*)
Age (years)					
<65 years	17	14	22.6	14.9–30.2	
≥65 years	8	8	17.3	0.0–35.0	0.308
Sex					
Female	13	11	17.3	3.5–31.1	
Male	12	11	23.2	16.5–29.8	0.524
Tumor location					
Head	22	20	22.1	15.6–28.7	
Others	3	2	7.0	0.1–14.1	0.622
Tumor stage					
pT1–2	13	11	22.6	15.6–29.5	
pT3–4	12	11	19.9	0.0–46.2	0.586
Differentiation					
Medium or high	16	14	19.9	10.8–29.0	
Low	7	6	6.6	0.0–16.3	0.689
Lymph node metastasis					
N0	9	8	23.2	0.0–70.3	
N1	16	14	19.9	10.8–29.0	0.847
Adjuvant chemotherapy					
No	18	17	17.3	0.0–38.7	
Yes	7	5	44.9	30.4–59.4	0.092

*pT* Pathologic evaluation of tumor specimen, *N0/N1* No presence/presence of regional lymph node metastasis. Adjuvant chemotherapy = postoperative gemcitabine treatment

To assess the relationships between p62 expression, clinicopathological variables, and survival, we performed univariable Cox proportional hazards regression analyses. The HR for death in patients with high p62 protein expression in tumor cells (when compared with low p62 protein expression in tumor cells) was 2.88 (1.17–7.11, *p* = 0.022; Table 4). In multivariable analysis (using a model that included p62, age, sex, tumor stage, lymph mode metastasis, differentiation, and adjuvant therapy), p62 was an independent prognostic factor for DSS **(**Table 4**)**.

**Table 4:** Univariable and multivariable Cox proportional hazard analyses of prognostic factors for disease-specific survival of patients with PDAC

Variable	Univariable analysis			Multivariable analysis^a^		
	HR	95% CI	*p*	HR	95% CI	*p*
Age						
< 65 years	1			1		
≥65 years	1.59	0.65–3.89	0.312	1.40	0.39–5.09	0.608
Sex						
Female	1			1		
Male	0.76	0.33–1.77	0.525	0.59	0.19–1.86	0.366
Tumor location						
Head	1					
Others	1.46	0.32–6.55	0.624		N.A.	
Tumor stage						
pT1–2	1			1		
pT3–4	1.26	0.55–2.92	0.587	1.15	0.30–4.37	0.839
Differentiation						
Medium or high	1			1		
Low	1.19	0.45–3.13	0.728	1.05	0.27–4.09	0.942
Lymph node metastasis						
N0	1			1		
N1	1.09	0.45–2.62	0.847	0.90	0.31–2.61	0.841
Adjuvant chemotherapy						
No	1			1		
Yes	0.42	0.15–1.18	0.101	0.37	0.10–1.34	0.130
p62						
Low	1			1		
High	2.88	1.17–7.11	**0.022**	3.83	1.24–11.84	**0.020**
LC3						
Low	1					
High	0.64	0.25–1.63	0.349		N.A.	
NRF2						
Low	1					
High	1.02	0.43–2.45	0.957		N.A.	

^a^Model including p62, age, sex, tumor stage, differentiation, lymph node metastasis, and adjuvant therapy. *HR* Hazard ratio, *CI* Confidence interval, *low* Staining grade low, *high* Staining grade high, *pT* Pathologic evaluation of tumor specimen, *N0/N1* No presence/presence of regional lymph node metastasis, *N.A*. Not analyzed. Adjuvant chemotherapy = postoperative gemcitabine treatment

**Fig. 5:**
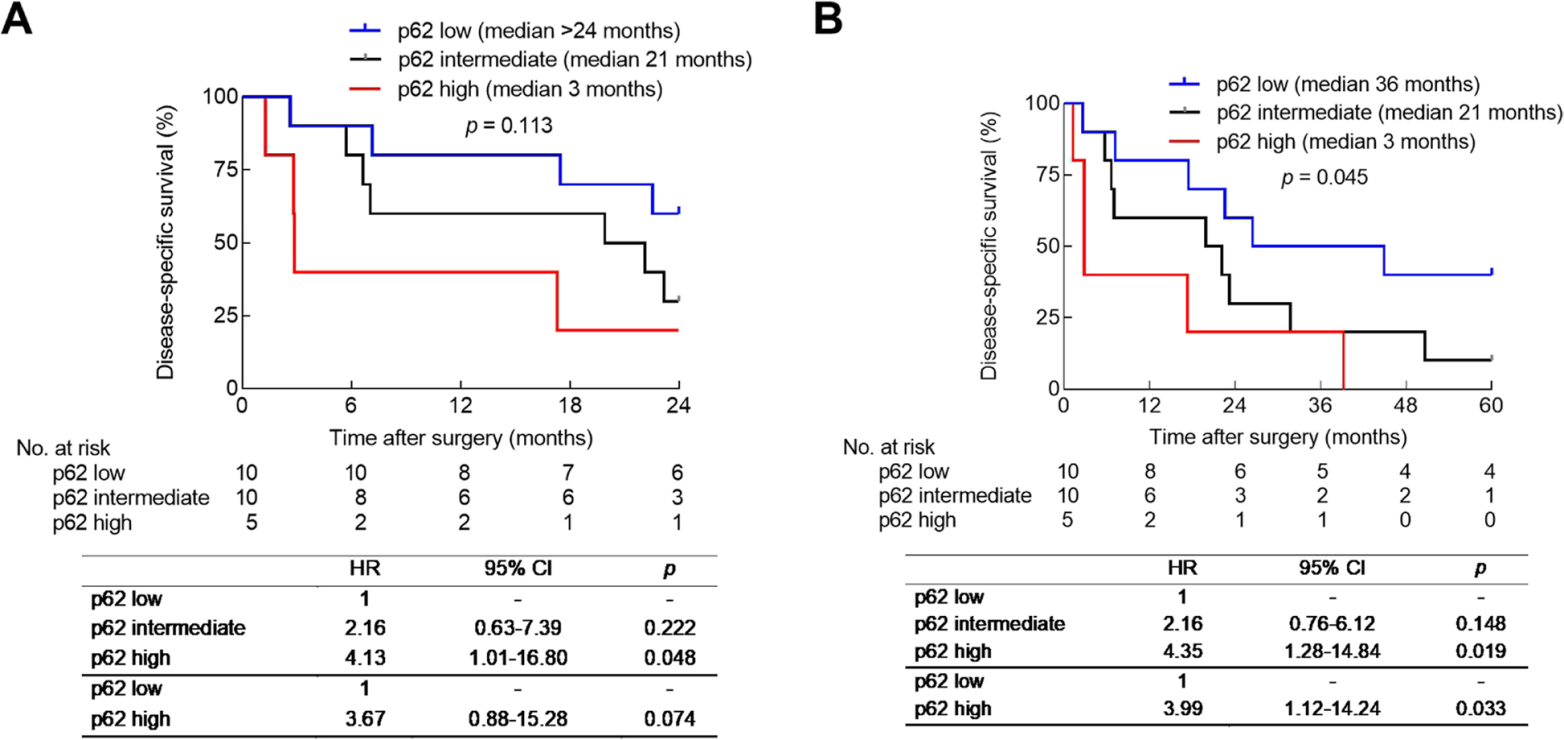
Kaplan–Meier disease-specific survival (DSS) curves and univariable Cox proportional hazard analyses for patients with low, intermediate and high tumor cell expression of p62. **A** 2-year survival among the 3 groups. **B** 5-year survival among the 3 groups

To assess the prognostic value of p62 expression in more detail, we used two staining score cutoffs to divide the patients into 3 groups (p62 low, p62 intermediate, and p62 high) and performed 2-year and 5-year DSS analyses using Kaplan-Meier curves and Cox proportional hazards regression analyses. Patients in the p62 high group tended to have shorter 2-year survival compared with patients in the p62 low group (HR: 3.67 [0.88–15.28], *p* = 0.074, Fig. 5A). Analysis of 5-year DSS showed that patients in the p62 high group had significantly shorter survival compared with patients in the p62 low group (HR: 3.99 [1.12–14.24], *p* = 0.033, Fig. 5B**).** Patients in the p62 high group had a prognosis comparable to the patients in the p62 intermediate group with a 2-year and 5-year DSS HR of 2.16 (0.63–7.39) and 2.16 (0.76–6.12), respectively (*p* = 0.222 and *p* = 0.148, respectively). In multivariable analysis (using a model that included p62, age, sex, tumor stage, lymph mode metastasis, differentiation, and adjuvant therapy), p62 expression level was not found to be an independent prognostic factor for DSS (2-year and 5-year DSS, p62 high vs p62 low: *p* = 0.125 and *p* = 0.129, respectively).

## Discussion

In this study, we show that high p62 protein expression predicts shorter survival of patients with resected PDAC, supporting a role for p62 as a prognostic marker in PDAC treated with primary surgery.

To the best of our knowledge, this study is the first to show an association between high expression of p62 protein in tumor cells and survival in patients with resected PDAC. Although previous studies have reported positive p62 staining in PDAC tissue [2, 10], none has shown a significant association with survival. One group reported that p62 staining was significantly stronger in PDAC than in normal tissue or tissue with pre-malignant pancreatic intraepithelial neoplasia lesions [2]. Another study showed positive p62 staining in a majority of PDAC cases without any significant association with clinicopathological variables or overall survival [10].

In contrast, recent experimental studies have provided better insight into the biology of p62-mediated signaling in PDAC. One group reported that accumulation of p62 in stressed pancreatic acinar cells harboring mutated *Kras* was associated with PDAC development in mice and maintenance of malignancy in human PDAC cells [2]. An earlier study showed that p62 supports *Kras*-induced inflammation, which promotes PDAC development in mice [1]. Together, these findings highlight a crucial role for p62 in KRAS-driven pancreatic tumorigenesis. Because most human PDACs carry mutations in *KRAS* [16], p62 accumulation and activation of downstream tumor-promoting signaling also likely play an important role in the pathogenesis and progression of human PDAC.

Our finding of no significant association between high LC3 protein in tumor cells and survival is in contrast with some reports of a link between LC3 staining and patient outcomes [17, 18]. One study showed that high LC3 staining in patient tumors was significantly associated with shorter survival and that LC3 is an independent prognostic factor for survival [18]. Another group similarly found a correlation of strong positive staining for LC3 overall and in the peripheral tumor area with poor patient outcome [17]. However, in agreement with our results, another study showed no significant association between LC3 and survival [19]. Furthermore, we found no significant association between survival and staining for NRF2, one of the possible downstream signaling pathways of p62. Only one previous study has indicated an association between increased nuclear NRF2 and poor survival [7].

Our results suggest that p62 is an independent prognostic factor for shorter DSS when follow up time was more than 5 years, at least in our small patient cohort. However, the small numbers of patients in this study may explain why we found no significant associations between well-established prognostic factors such as tumor differentiation, lymph node metastasis, and survival [20–22]. Other reasons for these discrepancies could be the use of different antibodies or in the evaluation of the immunohistochemical staining. We used a histoscore system, in which staining intensity and percentage of tumor cells showing positive staining were assessed separately before being combined into a staining score, in which relatively more weight was given to higher-intensity staining in a given tumor sample. In addition, in contrast to our study relying on whole tissue sections, some previous groups performed immunohistochemical analysis on tissue microarrays, which can have limitations in survival analyses of small numbers of patients and when the number of tumor cores is limited [23, 24].

Our finding of an association between high p62 protein expression in tumor cells and shorter survival among patients with PDAC treated with primary surgery may be highly relevant for ongoing clinical studies of autophagy inhibitors, such as chloroquine and hydroxychloroquine in adjuvant therapies combined with chemotherapy [25, 26]. Because p62 is a signaling adaptor protein and is itself degraded by autophagy, autophagy inhibition may increase p62 levels and thereby activate p62-mediated signaling [4, 27, 28] in tumor cells. Although the mechanisms for how high expression of p62 in tumor cells causes shorter survival remain unknown, our results suggest a need for ongoing and future clinical investigations of autophagy inhibitors to monitor p62 levels in PDAC tumor cells, as too high levels may be devastating for patients.

In conclusion, we found that high p62 protein expression in tumor cells is associated with shorter survival following pancreatic tumor resection. These results support a role for p62 as a prognostic marker in patients with PDAC treated with primary surgery.

## Supplementary Information

**Additional file 1.**

## Data Availability

All data generated or analyzed during this study are included in this published article.

## Abbreviations

PDAC: Pancreatic ductal adenocarcinoma
LC3: Microtubule-associated protein 1A/1B-light chain 3
NRF2: Nuclear factor erythroid 2-related factor 2
SQSTM1: Sequestosome-1
IHC: Immunohistochemistry
DAB: 3,3′-Diaminobenzidine
H-score: Histoscore
OS: Overall survival
HR: Hazard ratio
CI: Confidence interval
